# Genetic polymorphisms in ERCC1 and ERCC2 genes are associated with response to chemotherapy in osteosarcoma patients among Chinese population: a meta-analysis

**DOI:** 10.1186/s12957-017-1142-3

**Published:** 2017-04-07

**Authors:** Haiguang Zhang, Junbo Ge, Huanyu Hong, Lili Bi, Zhengwen Sun

**Affiliations:** 1 Yantaishan Hospital, No. 91 Jiefang Road, 264000 Yantai City, Shandong Province China; 2 Yeda Hospital, 264000 Yantai, Shandong Province China

**Keywords:** ERCC1, ERCC2, Chemotherapy response, Polymorphism, Meta-analysis

## Abstract

**Background:**

There existed controversies about the association between the response to chemotherapy for osteosarcoma (OS) patients and the genetic polymorphisms in excision repair cross-complementation group (ERCC1 and ERCC2) genes. We aimed to perform a meta-analysis to comprehensively evaluate the association.

**Method:**

We searched multiple databases for literature retrieval including the PubMED (1966 ∼ 2017), Embase (1980 ∼ 2017), and the Web of science (1945 ∼ 2017). The overall odds ratios(OR) and their corresponding 95% confidence interval (CI) were calculated for the three polymorphisms under the dominant, recessive, and allelic models.

**Results:**

From six eligible articles in our study, we found that for ERCC1 rs11615 polymorphism, a significant association was detected between the chemotherapy response and the polymorphism under all three models (dominant model: OR = 2.015, *P* = 0.005; recessive model: OR = 1.791, *P* = 0.003; allelic model: OR = 1.677, *P* = 0.003), and OS patients carrying C allele in rs11615 polymorphism were more likely to response to chemotherapy. In terms of ERCC2 rs1799793 polymorphism, this polymorphism was significantly associated with the response to chemotherapy for OS patients under recessive model (OR = 1.337, *P* = 0.036), and patients with AG + AA genotype in rs1799793 polymorphism were more appropriate to receive chemotherapy. With respect to ERCC2 rs13181 polymorphism, this polymorphism was not correlated with the response to chemotherapy for OS patients under all three models.

**Conclusions:**

Our meta-analysis suggested that among Chinese population, the rs11615 and rs1799793 polymorphisms were significantly correlated with the response to chemotherapy for patients with OS, and patients with CC or TC + CC genotypes in ERCC1 rs11615 polymorphism or AG + AA genotype in ERCC2 rs1799793 polymorphism were more suitable for chemotherapy.

## Background

Osteosarcoma, the most common primary malignancy of bone, is a devastating disease due to its rapid dissemination and poor prognosis [1–3]. Approximately 60% of OS sufferers are pediatric patients whose ages are ranged from 10 to 20 years old [4]. Patients with OS usually have some clinical symptoms characterized by pain and swelling in the affected bone, which is too intense and intolerable to wake them from sleep [5]. There are 15–30% of cases who have pulmonary metastasis when they are diagnosed as OS, which frequently results in patient death [6].

The current well-established strategy for the treatment of newly diagnosed OS was the combination of neoadjuvant chemotherapy, surgical resection for metastatic OS patients, and the adjuvant chemotherapy after surgery [5]. The neoadjuvant therapy for OS is the combination of cisplatin with doxorubicin, methotrexate, and ifosfamide, which contributes to an improved 5-year survival rate for patients without metastasis [7]. Among the chemotherapy agents, cisplatin, a platinum analog which can prevent cell division and growth by interfering with DNA, is a commonly used treatment for various kinds of tumors [8]. Moreover, accumulating large cooperative group studies and international collaboration have documented cisplatin as one of the ideal agents of the effective combined chemotherapy for OS treatment [5].

The nucleotide excision repair (NER) pathway, a highly powerful and sophisticated DNA damage removal pathway, has been believed to play important roles in cancer progression and response to platinum-based chemotherapy [7, 9]. Excision repair cross-complementation groups 1 (ERCC1) and 2 (ERCC2) are genes encoding two key enzymes in NER pathway [10]. It has been reported that single nucleotide polymorphisms (SNPs) of *ERCC1* and *ERCC2* genes are associated with the response to chemotherapy for OS. A retrospective study showed that ERCC2 rs1799793, a DNA repair polymorphism, was a predictive factor for chemotherapy response in OS patients [11]. A study published on 2015 suggested that the polymorphism of ERCC1 rs11615 affected on the response to chemotherapy in OS treatment [12]. However, there is debate on the predictive value of SNPs in *ERCC1* and *ERCC2* genes for the response to chemotherapy for OS. Study from Yang et al. found that polymorphism of ERCC1 rs11615 did not significantly influence the response to chemotherapy in patients with OS [13]. The ERCC2 rs1799793 polymorphism has been proved to not be associated with the response to chemotherapy for OS by a prospective study [14]. Herein, in order to comprehensively evaluate the association between the response to chemotherapy for OS patients and the SNPs in ERCC1 and ERCC2 genes including rs11615, rs1799793, and rs13181 polymorphisms, we pooled all related data together and performed the current meta-analysis.

## Methods

### Search strategy

The PubMED (1966 ∼ 2017), Embase (1980 ∼ 2017), and the Web of science(1945 ∼ 2017) were searched for study retrieval with a combination of Medical Subject Headings (MeSH) and text words relating to “ERCC1”, “ERCC2”, “osteosarcoma”, and “chemotherapy” as the search strategy. We retrieved literatures from the database inception to March 5th, 2017. The reference lists of identified articles and related reviews were examined to avoid any omission of eligible studies by the above electronic search strategy.

### Inclusion and exclusion criteria

In order to get more reliable estimations, we pre-defined strict inclusion criteria as follows: (1) all the participants were OS patients treated with chemotherapy; (2) detecting the relationship between the response to chemotherapy, and the SNPs in ERCC1 and ERCC2 genes such as rs11615, rs1799793, and rs13181 polymorphisms; (3) studies conducted on Chinese population; (4) providing available genotype data of relevant polymorphisms in ERCC1 and ERCC2 genes; (5) full-text studies published in English. Articles were eliminated if one of the following existed: (1) other SNPs rather than rs11615, rs1799793, and rs13181 polymorphisms; (2) SNPs of ERCC1 and ERCC2 genes are risk factors for OS survival; (3) literature types such as news, books, communications, letters, and reviews.

### Data extraction

The following data were collected independently from incorporated studies by two reviewers according to the mentioned inclusion and exclusion criteria: the first author, year of publication, number of patients, treatment approaches, genotyping methods, the age and gender ratio of patients, and genotyping data of ERCC1 rs11615, ERCC2 rs1799793, and ERCC2 rs13181 polymorphisms.

### Statistical analysis

We followed the Preferred Reporting Items for Systematic reviews and Meta-Analyses (PRISMA) guidelines [15]. In our study, the analyses were done with the STATA 12 software (STATACorp LP, College Station, TX, USA), and the value of *P* less than 0.05 was regarded as statistically significant. The pooled odds ratios (ORs) were calculated for dominant model, recessive model, and allelic model for ERCC1 rs11615, ERCC2 rs1799793, and ERCC2 rs13181 polymorphisms, respectively. The evaluation of response to chemotherapy was identified as the previous relevant studies [12, 16, 17]. An OR > 1 refers less OS patients with poor response to chemotherapy occur in the reference group, and patients in reference group have higher response rate to chemotherapy. We firstly used the Mantel-Haenszel (M-H) fixed-effects model to calculate the *I* ^2^ index as assessment of the heterogeneity among the incorporated studies. If the *I* ^2^ was less than 50%, we believed there was no significant heterogeneity and adopted the fixed-effects model to calculate the OR and its corresponding 95% CI. Otherwise, the DerSimonian and Laird (D-L) random-effects model was selected for the calculation of OR and 95% CI. Begg’s funnel plots were constructed to examine the publication bias. The noticeable asymmetry in the shape of funnel plot indicates publication bias. Egger’s tests were performed for further investigation, and the significance level was set at 0.05. The Rosenthal’s fail-safe numbers were calculated to estimate stability of the results [18]. The formula is as follows: *N*_fs0.05_ = (ΣZ/1.64)^2^−*n*, where *Z* is *Z* scores for the individual significance values, and *n* is the number of studies. A fail-safe number is often considered robust if it is greater than 5n + 10 [19].

## Results

### Study characteristics

**Fig. 1:**
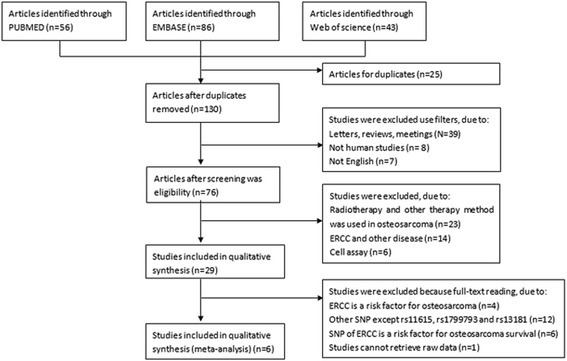
Flow diagram displaying study selection and specific reasons for exclusion from the mete-analysis

Based on our search strategy, we retrieved 56 literatures from PUBMED, 86 from Embase, and 43 from the Web of science. Twenty-five duplicated articles were removed, leaving 130 literatures for further assessment. After screening the titles, 54 literatures were eliminated. Then the remaining 76 articles were estimated for eligibility according to our inclusion and exclusion criteria. Finally six eligible articles [12–14, 17, 20, 21] were included in our meta-analysis. The flow diagram of study selection process and reasons for exclusion was represented in Fig. 1. Table 1 gives a summary of the characteristics of each included study.

**Table 1:** Summary of characteristics of studies in the meta-analysis

Study	Number of patients	Treatment approaches	Genotyping methods	Age	Male (%)
Z.H. Cao (2015)	186	Cisplatin-based chemotherapy	PCR-RFLP	19.2 ± 9.4	57.53
Z.F. Liu (2015)	115	Cisplatin-based chemotherapy	MALDI-TOF MS	–	56.52
Y.J. Sun (2015)	175	Chemotherapy	PCR-RFLP	17.8 ± 9.7	66.28
W.P. Ji (2015)	214	Cisplatin-based chemotherapy	PCR-RFLP	18.7 ± 11.5	62.15
Q. Zhang (2015)	260	Cisplatin-based chemotherapy	PCR-RFLP	18.4 ± 8.5	43.84
L.M. Yang(2012)	187	Neoadjuvant chemotherapy	PCR-RFLP	17.7 ± 9.6	56.68

*PCR-RFLP* polymerase chain reaction restriction fragment length polymorphism assay, *MALDI-TOF MS* matrix-assisted laser desorption/ionization time-of-flight mass spectrometry method, – unavailable

### The relationship between the response to chemotherapy for OS patients and the ERCC1 rs11615 polymorphism

**Fig. 2:**
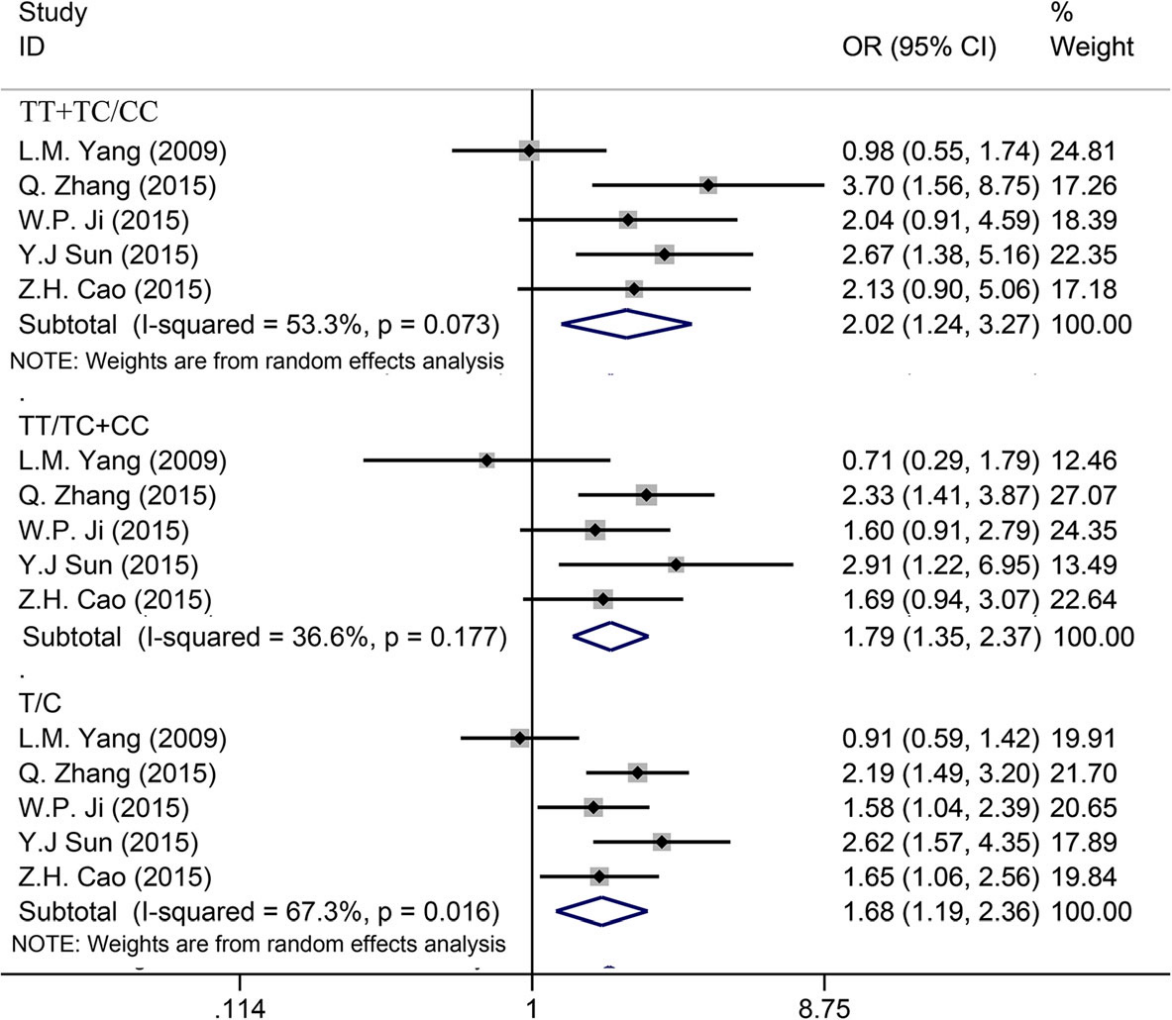
Forest plot of study evaluating the relationship between the response to chemotherapy for OS patients and the ERCC1 rs11615 polymorphism

Five studies including 1019 OS patients were incorporated to evaluate the association of the rs11615 polymorphism and the response to chemotherapy. The results could be found in Table 2. For dominant (TT + TC versus CC) and allelic models (T versus C), the random effects model was chosen to calculate the OR and 95% CI due to the large heterogeneity. The ORs for TT + TC versus CC and T versus C were 2.015 and 1.677, respectively (TT + TC versus CC: 95% CI:1.242–3.271, *P* = 0.005, Fig. 2; T versus C: 95% CI: 1.194–2.356, *P* = 0.003, Fig. 2), which suggested that significant association was detected between the rs11615 polymorphism and the response to chemotherapy for OS patients under the dominant and allelic models, and there were more responders to chemotherapy in patients with CC genotype in rs11615 polymorphism. With regard to the recessive model (TT versus CC + TC), considering the small heterogeneity, we selected the fixed-effects model to yield the OR for this model. The OR for TT versus CC + TC was 1.791 (95% CI:1.353–2.372, *P* = 0.003, Fig. 2), revealing that the rs11615 polymorphism was significantly associated with the response to chemotherapy for OS patients under recessive model, and patients with CC + TC genotype in rs11615 polymorphism had higher response rate to chemotherapy.

**Table 2:** Meta-analysis of the association of the response to chemotherapy for OS patients and the rs11615, rs1799793, and rs13181 polymorphisms

SNP	Total patients (*n*)	Model	OR	Lower limit	Upper limit	*P* (OR)	*I* ^2^ (%)	*P* (Heterogeneity)	*P* (Begg’s test)	*P* (Egger’s test)	FSN
rs11615	1019	Dominant model (TT + TC/CC)	2.015	1.242	3.271	0.005	53.30	0.073	0.462	0.168	25.000
		Recessive model(TT/TC + CC)	1.791	1.353	2.372	0.003	36.60	0.177	0.462	0.508	22.393
		Allelic model(T/C)	1.677	1.194	2.356	0.003	67.30	0.016	0.806	0.927	105.107
rs13181	1133	Dominant model (AA + AC/CC)	1.058	0.739	1.516	0.757	<0.01	0.572	0.707	0.511	3.434
		Recessive model(AA/AC + CC)	1.135	0.868	1.484	0.355	16.10	0.31	0.26	0.079	3.160
		Allelic model(A/C)	1.091	0.894	1.331	0.392	37.40	0.157	0.452	0.271	14.151
rs1799793	949	Dominant model (GG + GA/AA)	1.54	0.982	2.413	0.06	22.30	0.272	0.221	0.317	44.628
		Recessive model(GG/AG + AA)	1.337	1.019	1.754	0.036	36.70	0.177	1.000	0.98	56.223
		Allelic model(G/A)	1.328	0.943	1.87	0.105	57.60	0.051	1.000	0.973	92.857

*n* number of participants, *FSN* fail-safe numbers

### The relationship between the response to chemotherapy for OS patients and the ERCC2 rs13181 polymorphism

**Fig. 3:**
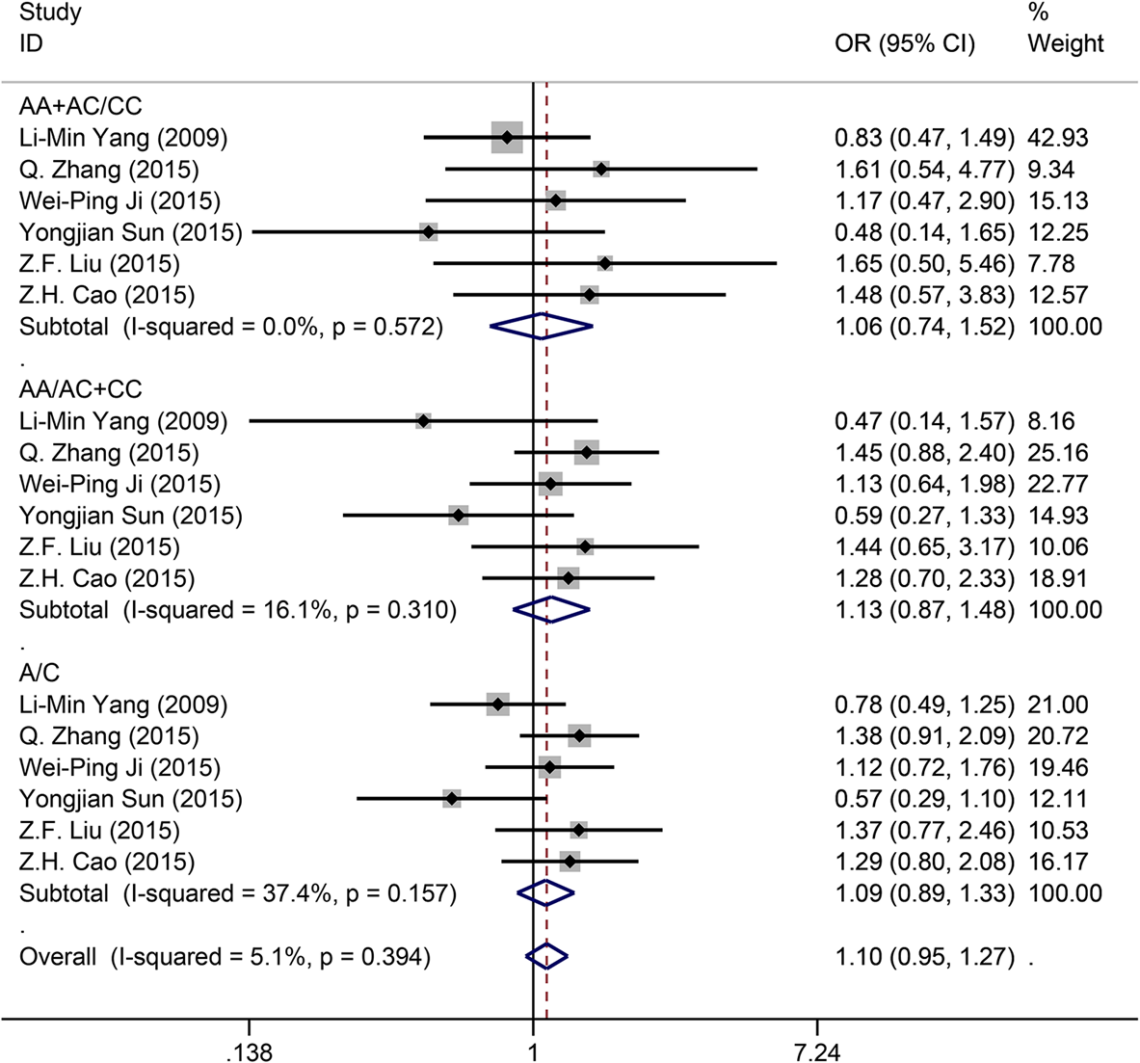
Forest plot of study assessing the relationship between the response to chemotherapy for OS patients and the ERCC2 rs13181 polymorphism

All eligible studies together pooled 1133 OS patients involved in the association of the response to chemotherapy and the rs13181polymorphism. Table 2 gave a summary of the results. The values of *I* ^2^were lower than 50% for all the three models, so the fixed-effects model was used to achieve the OR and 95% CI. The ORs for dominant model (AA + AC versus CC), recessive model (AA versus AC + CC), and allelic model (A versus C) were 1.058 (95% CI: 0.793–1.516, *P* = 0.757, Fig. 3), 1.135 (95% CI: 0.868–1.484, *P* = 0.355, Fig. 3) and 1.091 (95% CI: 0.894–1.331, *P* = 0.392, Fig. 3), respectively, which demonstrated that no significant association was found between the rs13181 polymorphism and the response to chemotherapy for OS patients under all the three models.

### The relationship between the response to chemotherapy for OS patients and the ERCC2 rs1799793 polymorphism

**Fig. 4:**
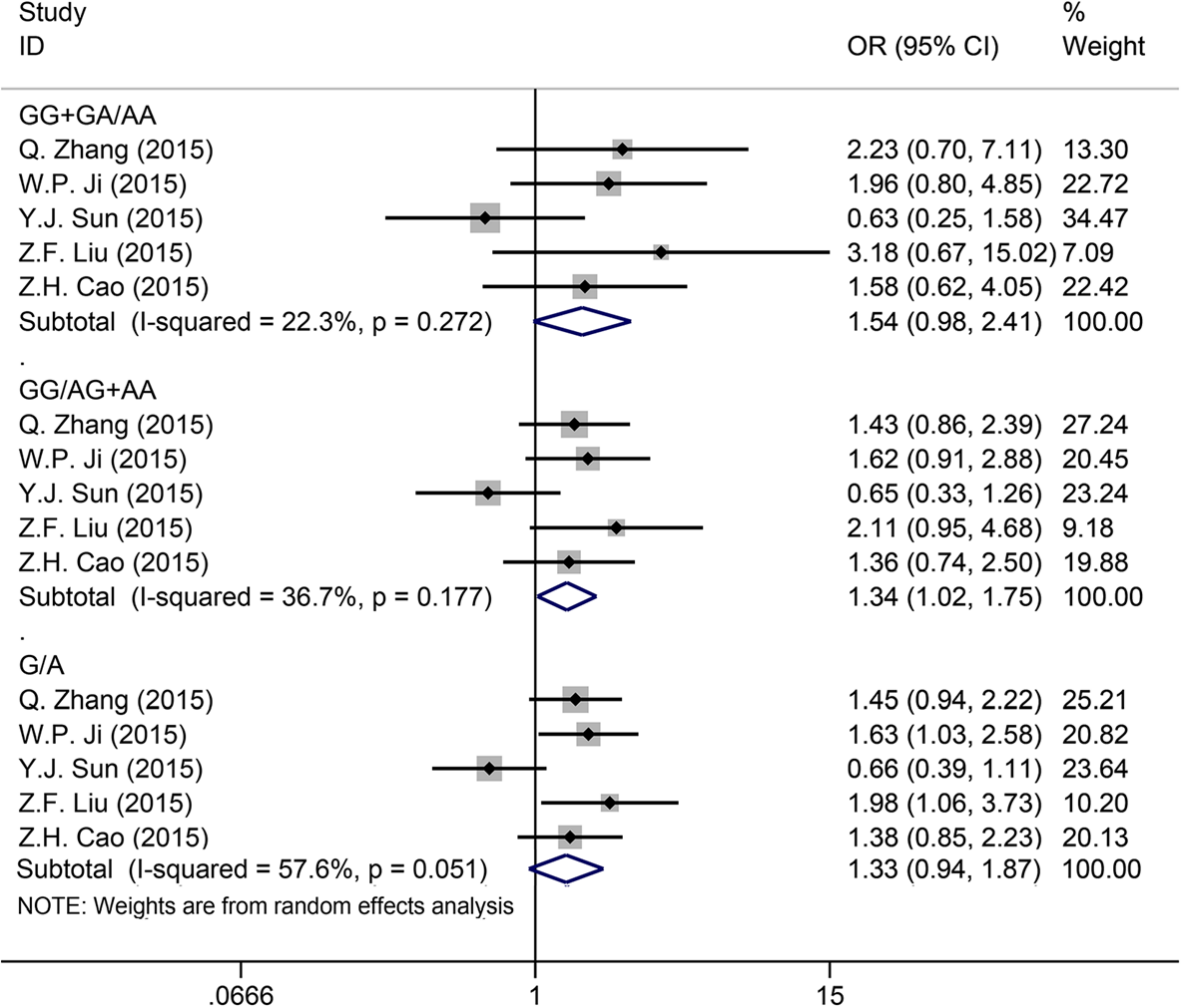
Forest plot of study estimating the relationship between the response to chemotherapy for OS patients and the ERCC2 rs1799793 polymorphism

There were five eligible studies for the analysis of association between the rs1799793 polymorphism and the response to chemotherapy for OS patients. The results were displayed in Table 2. For the dominant (GG + GA versus AA) and recessive models (GG versus AG + AA), the fixed-effects model was adopted for the estimation of OR and 95% CI. The ORs for GG + GA versus AA and GG versus AG + AA were 1.54 (95% CI: 0.982–2.413, *P* = 0.06, Fig. 4) and 1.337 (95% CI: 1.019–1.754, *P* = 0.036, Fig. 4), respectively, suggesting that the rs1799793 polymorphism was significantly associated with the response to chemotherapy for OS patients under recessive model, and patients with GG genotype in rs179973 polymorphism had poor response to chemotherapy. In terms of the allelic model, the *I* ^2^ was 57.60%, so the random effects model was applied for yielding the OR and 95% CI. The OR for G versus A was 1.328 (95% CI: 0.943–1.87, Fig. 4), and the *P* was higher than 0.05, which implied that no significant association was observed under allelic model.

### Publication bias

**Fig. 5:**
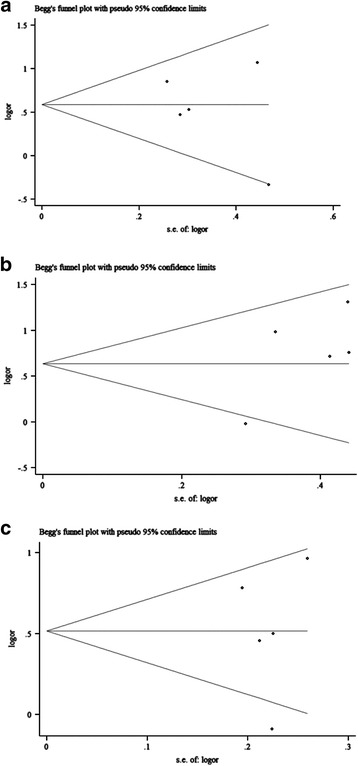
Funnel plots of studies with ERCC1 rs11615 polymorphism under dominant model (**a**), recessive model (**b**), and allelic model (**c**)

**Fig. 6:**
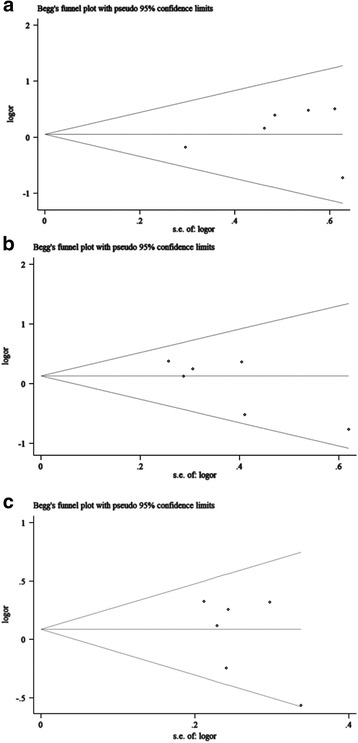
Funnel plots of studies with ERCC2 rs13181 polymorphism under dominant model (**a**), recessive model (**b**), and allelic model (**c**)

**Fig. 7:**
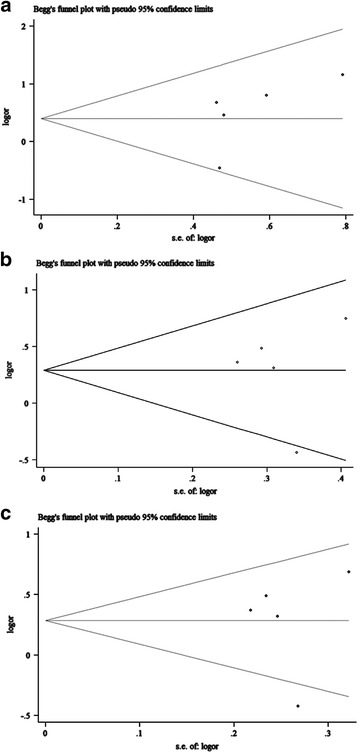
Funnel plots of studies with ERCC2 rs1799793 polymorphism under dominant model (**a**), recessive model (**b**), and allelic model (**c**)

We observed no obvious asymmetry in the shape of funnel plots (Figs. 5, 6 and 7), referring that there was no significant publication bias in the analyses. Moreover, all the values of *P* in both Begg’s and Egger’s test were higher than 0.05, which further provided evidence of no publication bias in our study. Rosenthal’s fail-save number suggested that the results in allelic model of rs11615 groups and all three rs1799793 groups were comparatively reliable. However, the publication bias could not be ignored in dominant model and recessive model of rs11615 groups, and all three rs13181 groups (Table 2).

## Discussion

In the current study, we performed a meta-analysis to assess the association of the response to chemotherapy for OS patients and the rs11615, rs1799793 and rs13181 polymorphisms. Our results showed that for ERCC1rs11615, OS patients carrying CC or TC + CC genotypes were more likely to respond to the chemotherapy, and patients with C allele in rs11615 polymorphism were more appropriate to receive chemotherapy. With respect to ERCC2 rs1799793, the response rate to chemotherapy in patients with AG + AA genotype was significantly higher than that in patients carrying GG genotype, and patients carrying AG + AA genotype in rs1799793 polymorphism were more suitable for chemotherapy, while for ERCC2 rs13181, the polymorphism had a null effect on the response to chemotherapy for OS patients.

Despite there is only 3 cases of OS per 1,000,000 individuals, OS is the most primary malignant of bone tumor worldwide occupying approximately 56% of all bone tumors [22, 23]. It has been reported that OS arises from mesenchymal cells undergoing abnormal modifications during the differentiation progress, which leads OS to a heterogenic tumor [4]. With the usage of preoperative and postoperative chemotherapy in clinic, the prognosis of OS without metastasis has been improved obviously, and the 5-year overall survival rate is elevated to 77%, while the prognosis for patients with metastatic OS is poor with a lower (less than 20%) 5-year overall survival rate due to its resistance to conventional chemotherapy [24]. Not only factors such as the age, gender, and ethnicity impact on the incidence of OS, but also genetic polymorphisms including CTLA-4 + 49A/G and TGF-β1 29 T/C variants are reported to be significantly correlated with OS susceptibility [25].

ERCC1, located at 19q13, encodes a rate-limiting enzyme in NER pathway, which can repair chemical drug-induced DNA damage [26, 27]. The rs11615, one of the common polymorphisms in ERCC1, may decrease the expression of ERCC1 mRNA, thus reducing the resistance to chemotherapy for cancer patients [26]. Cancer cells overexpressing ERCC1 were correlated with drug resistance to chemotherapy containing cisplatin, carboplatin, or oxaliplatin in several types of tumors such as gastric, bladder, ovarian, colorectal, and lung carcinomas [28]. Zhang et al. observed that the *ERCC1* rs11615 polymorphism might influence the clinical outcomes and response to chemotherapy for patients with OS, and patients with CC genotype in *ERCC1* rs11615 polymorphism had better response to chemotherapy [20] which was also confirmed in our meta-analysis, we furthermore incorporated all relevant data together and considered OS patients with CC or TC + CC genotypes had better response to chemotherapy. Thus, the genetic polymorphism of rs11615 is a potentially alternative target for OS patients in clinical diagnosis, and the C allele in *ERCC1* rs11615 polymorphism for patients with OS could be an underlying candidate predictor in clinical chemotherapy treatment.

ERCC2, located at 9q13.3, encodes a DNA helicase which causes repair of single-strand DNA injury [29]. ERCC2 gene possesses more than 500 SNPs, among which rs13181 and rs1799793 are the two common polymorphisms that can alter the amino acid sequence in the ERCC2 gene [30]. The ERCC2 rs13181 polymorphism has been documented to be associated with a higher susceptibility to glioma among the Chinese population [31]. And the ERCC2 rs1799793 polymorphism, together with ERCC1 rs11615 polymorphism, may play roles in the response to chemotherapy and overall survival for patients with gastric cancer [32]. As to the polymorphism in ERCC2 for OS patients, Liu et al. found that the rs1799793 polymorphism in *ERCC2* gene was likely to influence the chemotherapy response, and OS patients with AA genotype in *ERCC2* rs1799793 were likely to have better chemotherapy response, whereas the rs13181 polymorphism was not associated with the chemotherapy response [17], which was not exactly the same as our results. We indicated that the polymorphism of rs13181 in *ERCC2* gene was also not correlated with the response to chemotherapy for patients with OS. However, when enlarging the sample size by pooling all related data together in our meta-analysis, we detected that OS patients with AG + AA genotype of rs1799793 polymorphism were more likely to have good chemotherapy response which suggested AG + AA genotype could be a potential predictor for OS patients in clinical diagnosis and chemotherapeutic treatment. What we found were significant supplements to the molecular mechanism research of OS and will greatly benefit the OS patients in the future.

During the study retrieval, we found nine papers regarding the relationship between the chemotherapy response and the rs11615, rs1799793, and rs13181 polymorphisms in ERCC1 and ERCC2 genes for OS patients. Considering the different genetic and nationality backgrounds, studies conducted on Spanish [33], Slovenian [34] and Italian [7] populations were excluded, and only studies related to Chinese population were included and incorporated to get a more reliable and precise evaluation of the association between the response to chemotherapy and the polymorphisms of rs11615, rs1799793, and rs13181 in ERCC1 and ERCC2 genes for patients with OS among Chinese population.

To our knowledge, the present study is the first meta-analysis to explore the correlation of the chemotherapy response and the rs11615, rs1799793, and rs13181 polymorphisms in ERCC1 and ERCC2 genes for OS patients among Chinese population. However, there are some limitations in the current study. Firstly, as we mentioned above, there is only one article assessing the association between the response to chemotherapy and the polymorphisms of rs11615, rs1799793, and rs13181 for OS patients for three different nationalities including Spanish, Slovenian, and Italian, respectively. Although, we performed our meta-analysis on Chinese population with six eligible studies incorporated, with more studies becoming available for other nationalities, the overall meta-analysis and subgroup analysis stratified by nationalities would be conducted. Secondly, the genotyping methods in the included studies were not exactly the same, which might cause bias in our meta-analysis. Additionally, unpublished articles were not considered in our study.

## Conclusions

The current meta-analysis suggested that for OS patients among Chinese population, the rs11615 and rs1799793 polymorphisms were significantly associated with the chemotherapy response, and patients with CC or TC + CC genotypes in ERCC1 rs11615 polymorphism or AG + AA genotype in ERCC2 rs1799793 polymorphism were more likely to have good response to chemotherapy. These SNPs may be candidate pharmacogenomic factors capable of indentifying OS patients who are more appropriate to receive chemotherapy.

## Abbreviations

CI: Confidence interval
ERCC1: Excision repair cross-complementation group 1
NER: Nucleotide excision repair
OR: Odds ratios
OS: Osteosarcoma
SNPs: Single nucleotide polymorphisms
